# Malignant Hypertension and Torsades De Pointes as Initial Presentations of Primary Aldosteronism

**DOI:** 10.1016/j.jaccas.2026.106868

**Published:** 2026-02-09

**Authors:** Yanqiu Li

**Affiliations:** Department of Cardiology, The Third Affiliated Hospital of Chongqing Medical University (Fangda Hospital), Chongqing, China

**Keywords:** adrenal venous sampling, hypokalemia, malignant hypertension, primary aldosteronism, torsades de pointes

## Abstract

**Background:**

Primary aldosteronism (PA) is a common yet underdiagnosed cause of secondary hypertension, particularly in young patients presenting with severe disease.

**Case Summary:**

A 31-year-old woman presented with malignant hypertension (264/144 mm Hg), severe hypokalemia (K^+^ level 2.60 mmol/L), and acute pulmonary edema. Her initial work-up during nicardipine and furosemide infusion showed a normal aldosterone-to-renin ratio (ARR), leading to misdiagnosis as essential hypertension. Over a 3-year follow-up, she developed refractory hypertension during pregnancy and preeclampsia. She was readmitted with torsades de pointes triggered by severe hypokalemia (K^+^ level 1.90 mmol/L). Repeat testing revealed a markedly elevated ARR (667.26). Adrenal venous sampling confirmed right-sided PA. Right adrenalectomy confirmed a cortical adenoma, with subsequent normalization of blood pressure and potassium levels.

**Discussion:**

This case report highlights the interference of common medications in PA diagnosis, delineates the complete pathophysiological cascade from aldosterone excess to life-threatening arrhythmia, and reveals the dynamic nature of PA-associated adrenal nodules.

**Take-Home Messages:**

Clinicians must be vigilant about the confounding effects of common antihypertensive agents on ARR interpretation and should consider adrenal imaging as part of the dynamic management of PA. Adrenal venous sampling remains paramount for surgical planning.

## Graphical Abstract

**Figure:**
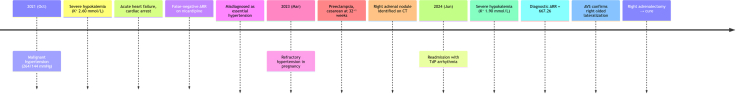

## History of Presentation

A 31-year-old woman presented to the emergency department with progressive cough and dyspnea. One month earlier, she was found to have elevated blood pressure (up to 220/140 mm Hg) but received no treatment. Physical examination revealed tachycardia (heart rate 159 beats/min), tachypnea (respiratory rate 38 breaths/min), a blood pressure of 264/144 mm Hg, and an oxygen saturation of 86%.

**Visual Summary:**
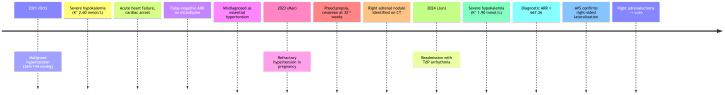
Case Timeline: Diagnostic and Therapeutic Journey ARR = aldosterone-to-renin ratio; AVS = adrenal venous sampling; CT = computed tomography; TdP = torsades de pointes.

## Past Medical History

The patient had no significant past medical history and no family history of hypertension.

## Differential Diagnosis

Initial differential diagnoses included essential hypertension with hypertensive emergency, primary aldosteronism (PA), other secondary causes of hypertension (renal artery stenosis and pheochromocytoma), and acute myocarditis.

## Investigations

**Figure 1:**
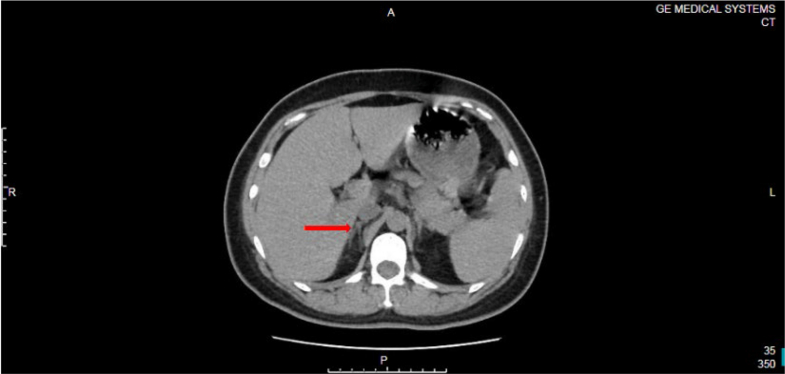
Noncontrast Chest Computed Tomography Scan From October 29, 2021, Incidentally Capturing the Adrenal Region (Arrows), Showing no Definite Nodule

Arterial blood gas analysis revealed metabolic acidosis with severe hypokalemia (K^+^ level 2.60 mmol/L). Chest radiography confirmed pulmonary edema. The N-terminal pro–B-type natriuretic peptide level was elevated to 1,333.58 pg/mL. The initial etiological work-up for hypertension, conducted during intravenous infusion of nicardipine and furosemide, showed that the aldosterone level was 240.40 pg/mL and the renin level 15.03 pg/mL (aldosterone-to-renin ratio [ARR] 15.99). Adrenal ultrasonography showed no abnormalities. A chest computed tomography (CT) scan incidentally captured portions of the adrenal glands but identified no definite nodules (Figure 1). The secretion rhythms of catecholamines and aldosterone were normal.

**Figure 2:**
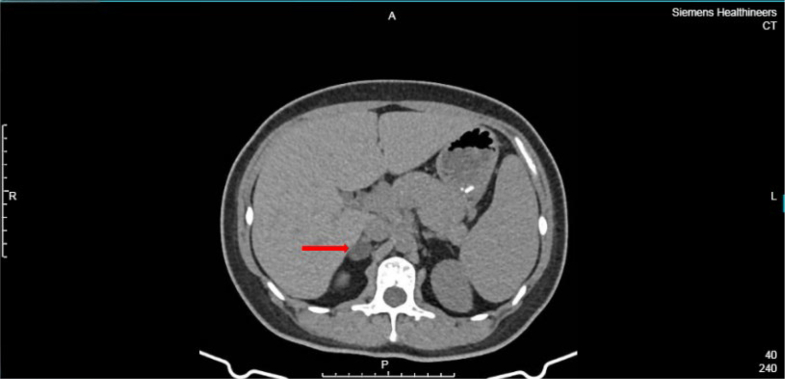
Contrast-Enhanced Adrenal Computed Tomography Scan From January 15, 2024, Revealing an Approximately 19 × 14 mm Nodule in the Right Adrenal Gland (Arrow)

During pregnancy (in 2023), persistent hypokalemia (K^+^ level 2.66 mmol/L) was documented. Postpartum CT identified a 19 × 14 mm right adrenal nodule (Figure 2).

**Figure 3:**
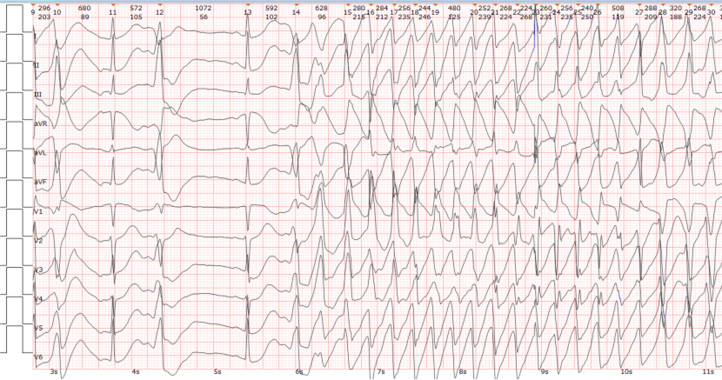
Rhythm Strip Recorded During Severe Hypokalemia (K^+^ Level 1.90 mmol/L), Showing an Episode of Torsades de Pointes

At readmission (in 2024), severe hypokalemia (K^+^ level 1.90 mmol/L) and multiple episodes of torsades de pointes (TdP) were documented (Figure 3). Repeat hypertensive evaluation demonstrated that the aldosterone level was 2,227.87 pg/mL and the renin level 0.34 pg/mL (ARR 667.26). Adrenal venous sampling (AVS) confirmed right-sided lateralization.

## Management

Initial management included cardiopulmonary resuscitation, endotracheal intubation, and intensive care unit admission for acute heart failure. After the initial admission, the patient was discharged on multiple antihypertensive agents and potassium supplements.

During pregnancy, she received nifedipine, labetalol, and methyldopa but blood pressure control remained inadequate, necessitating cesarean delivery at 32^+1^ weeks for preeclampsia.

Definitive management involved right adrenalectomy after confirmation of right-sided PA by AVS.

## Outcome and Follow-Up

Postoperatively, the patient's blood pressure control improved significantly, requiring only 2 oral antihypertensive agents. Potassium levels normalized without supplementation. No further arrhythmic events occurred during follow-up.

## Discussion

This case provides 3 inter-related core insights for cardiovascular practice.

### The pitfall of pharmacological interference in ARR diagnosis

This case is a classic example of medication interference in biochemical testing. The initial false negative ARR was a direct pharmacological consequence of nicardipine administration. Dihydropyridine calcium-channel blockers potently stimulate renin release via mechanisms including renal arteriolar vasodilation.^1^ In this patient, nicardipine artificially elevated renin from the suppressed levels typical of PA into the normal range whereas aldosterone remained inappropriately high for the context of hypertension with hypokalemia, mathematically depressing the ARR and creating a diagnostic blind spot.

It is critical to recognize that calcium-channel blockers are not the only antihypertensive agents that confound the ARR. Thiazide diuretic agents stimulate renin secretion via volume contraction and can induce hypokalemia, which itself suppresses aldosterone secretion.^2^ This combination of “elevated renin and suppressed aldosterone” severely distorts the ARR. Therefore, a comprehensive medication review is imperative before ARR testing.

### The dynamic nature of PA and insights from imaging evaluation

Imaging follow-up in this case offers a unique perspective on the natural history of adrenal nodular lesions. Initial adrenal ultrasound and limited chest CT at first presentation showed no definite nodules, whereas a CT scan 3 years later clearly revealed a right adrenal nodule (19 × 14 mm). This dynamic evolution “from absence to presence” strongly suggests that this aldosterone-secreting adenoma likely developed and grew over time.

This reveals that PA is not always a static condition and that functional adenomas have the potential to progress.^3^ This finding explains why early imaging may have negative results and underscores the necessity of repeat targeted adrenal imaging (eg, dedicated thin-section adrenal CT) during follow-up for patients with high clinical suspicion of PA despite initially negative imaging results. Although ultrasonography is convenient, its detection rate is highly operator dependent and influenced by patient body habitus, whereas CT offers higher spatial resolution and objectivity and is the imaging modality of choice for morphological assessment per guidelines.^4^

### The pathophysiological cascade from aldosterone excess to TdP

This case perfectly illustrates the mechanistic pathway from autonomous aldosterone overproduction to life-threatening ventricular arrhythmia. Aldosterone acts on mineralocorticoid receptors in principal cells of the distal nephron, up-regulating epithelial sodium channels and basolateral sodium-potassium adenosine triphosphatase, promoting potassium excretion and leading to hypokalemia.

Severe hypokalemia alters cardiac potassium channel function, notably causing voltage-dependent inactivation of the rapidly activating delayed rectifier potassium current. Inhibition of this major repolarizing current disrupts the balance between depolarizing and repolarizing forces during the action potential, leading to QT interval prolongation on the surface electrocardiogram. Furthermore, hypokalemia does not uniformly affect all myocardial layers; it preferentially prolongs the action potential duration of mid-myocardial M cells, thereby increasing transmural dispersion of repolarization, creating a vulnerable substrate within the ventricular myocardium. The prolonged repolarization phase also facilitates the occurrence of early afterdepolarizations. When an early afterdepolarization falls within the heterogeneous window of repolarization created by increased transmural dispersion of repolarization, it can precipitate TdP.^5^^,^^6^ Therefore, any unexplained QT interval prolongation or TdP should prompt an immediate search for reversible secondary causes, including PA.

### The imperative of adrenal venous sampling

Anatomical presence does not invariably correlate with hormonal hypersecretion.^7^ Guidelines recommend AVS for patients with confirmed PA who are surgical candidates. This case reinforces its necessity. Despite the clear identification of an adrenal nodule on CT, AVS was crucial for confirming it as the functional source. This is critical because if the nodule had been a nonfunctioning incidentaloma, a potentially noncurative surgery could have been avoided. AVS remains indispensable for confirming the functional source and guiding surgical lateralization, which is consistent with current guidelines.^8^

## Conclusions

This case demonstrates that PA can present with malignant hypertension and fatal arrhythmias. It underscores the critical importance of vigilance for medication interference when interpreting the ARR, reveals the dynamic, progressive nature of PA as a disease entity, and delineates the complete, reversible pathophysiological pathway from hormonal excess to end-organ damage.

### Patient Consent

Informed consent for publication was obtained from the patient's family.

**Take-Home Messages**

• Diagnostic vigilance: Primary aldosteronism should be highly suspected in young patients with severe or refractory hypertension and hypokalemia, even if the initial aldosterone-to-renin ratio appears “normal” because of interference from calcium-channel blockers or diuretic agents.
• Dynamic management perspective: Primary aldosteronism is a progressive disease, and associated adrenal nodules may evolve over time. Repeat targeted adrenal imaging during follow-up should be considered for cases with persistent clinical suspicion despite initially negative imaging results.

## Funding Support and Author Disclosures

This work was supported by the Internal Research Fund of The Third Affiliated Hospital of Chongqing Medical University (grant number KY24044). The authors have reported that they have no relationships relevant to the contents of this paper to disclose.
